# Supplementary material for: Torenia sp. Extracts Contain Multiple Potent Antitumor Compounds with Nematocidal Activity, Triggering an Activated DNA Damage Checkpoint and Defective Meiotic Progression
Source: Pharmaceuticals (Basel). 2024 May 10;17(5):611. doi: 10.3390/ph17050611 (PMC11124231; doi:10.3390/ph17050611)
Supplement: Supplementary file 1 [file pharmaceuticals-17-00611-s001.zip › pharmaceuticals-2991042-supplementary.pdf]

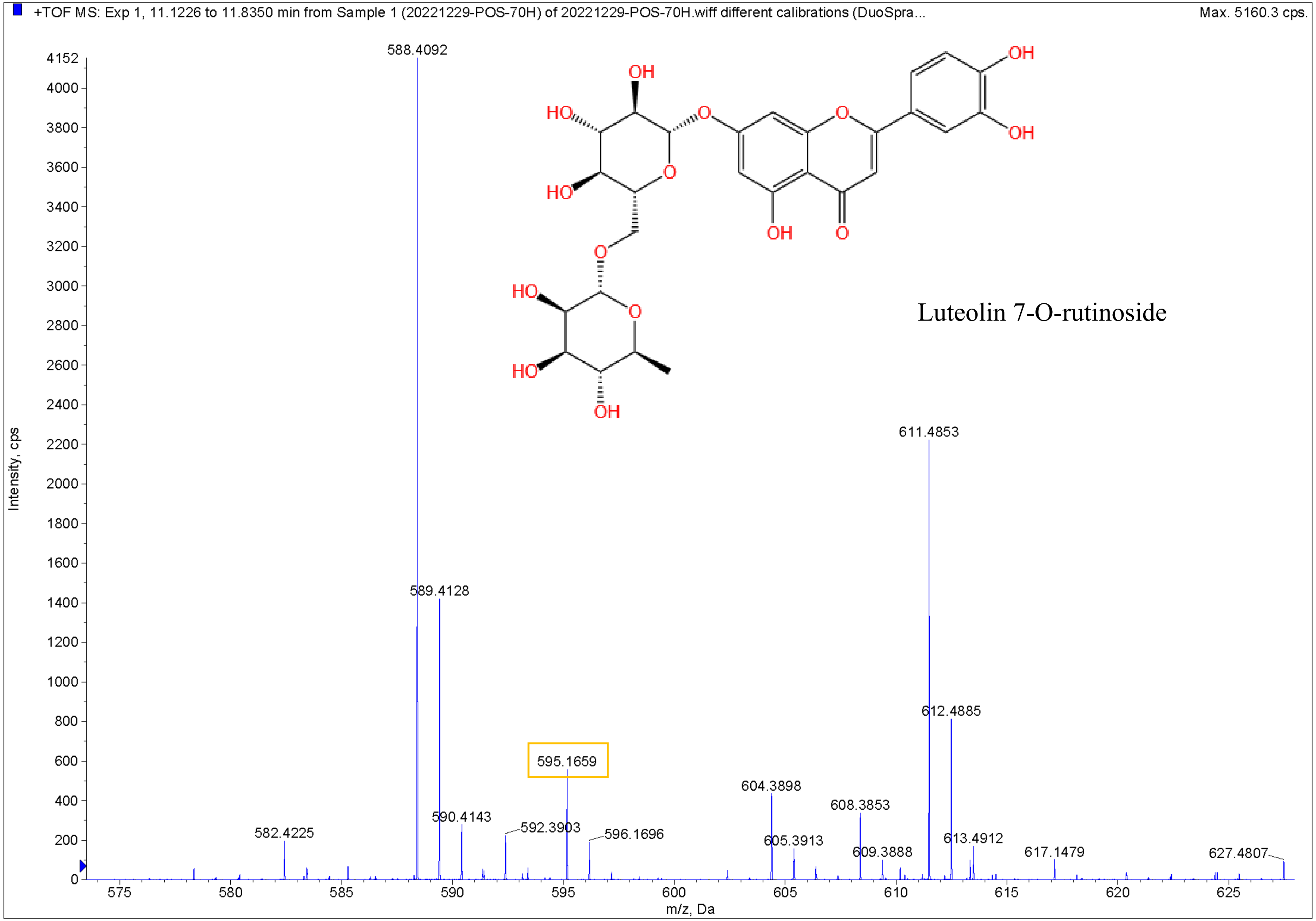
+TOF MS: Exp 1, 11.1226 to 11.8350 min from Sample 1 (20221229-POS-70H) of 20221229-POS-70H.wiff different calibrations (DuoSpra...
Max. 5160.3 cps.
Luteolin 7-O-rutinoside
Intensity, cps
m/z, Da
588.4092
589.4128
590.4143
592.3903
595.1659
596.1696
582.4225
604.3898
605.3913
608.3853
609.3888
611.4853
612.4885
613.4912
617.1479
627.4807

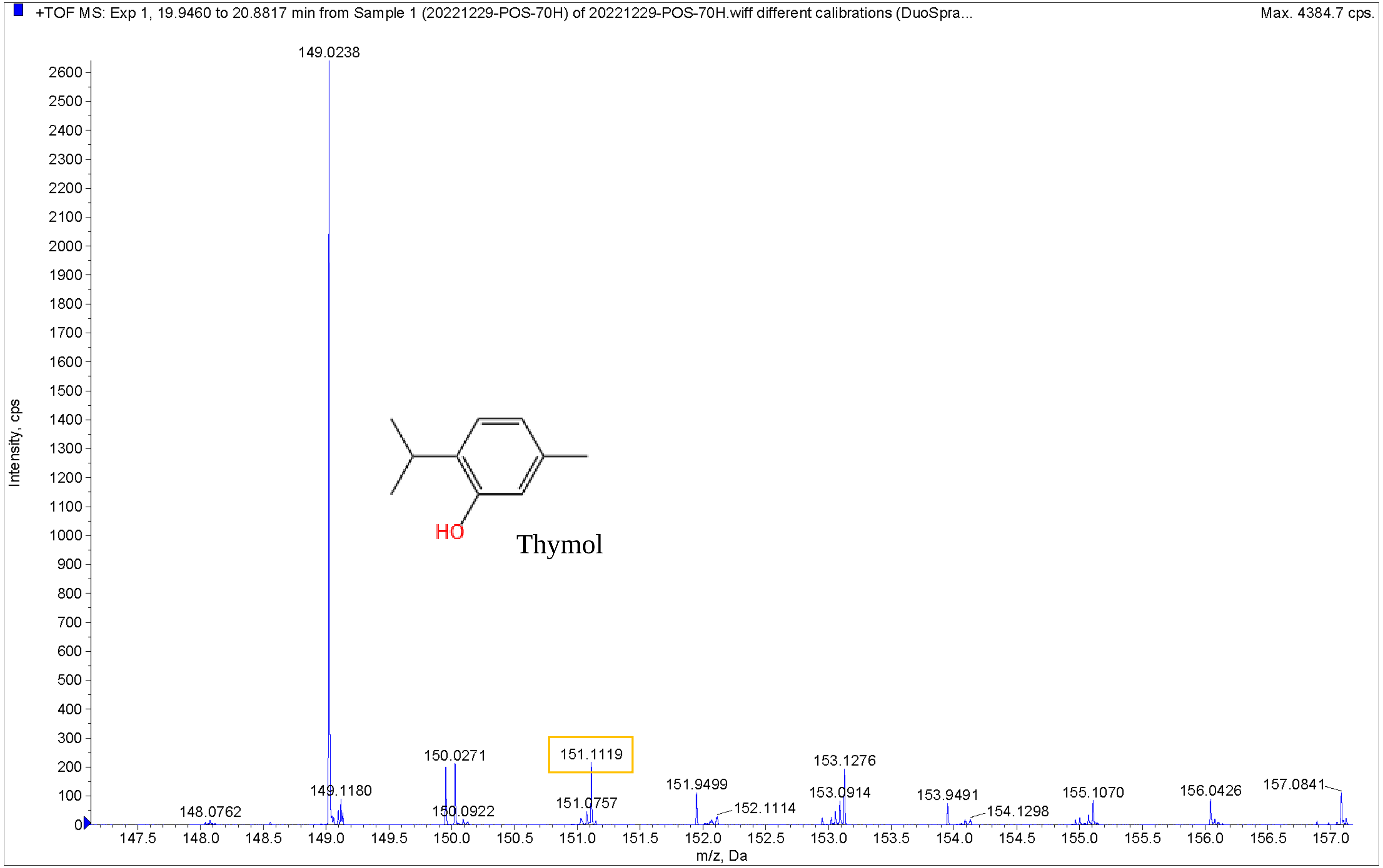

+TOF MS: Exp 1, 19.9460 to 20.8817 min from Sample 1 (20221229-POS-70H) of 20221229-POS-70H.wiff different calibrations (DuoSpra...
Max. 4384.7 cps.
Intensity, cps
m/z, Da
149.0238
149.1180
148.0762
150.0271
150.0922
151.1119
151.0757
151.9499
152.1114
153.1276
153.0914
153.9491
154.1298
155.1070
156.0426
157.0841
HO
Thymol

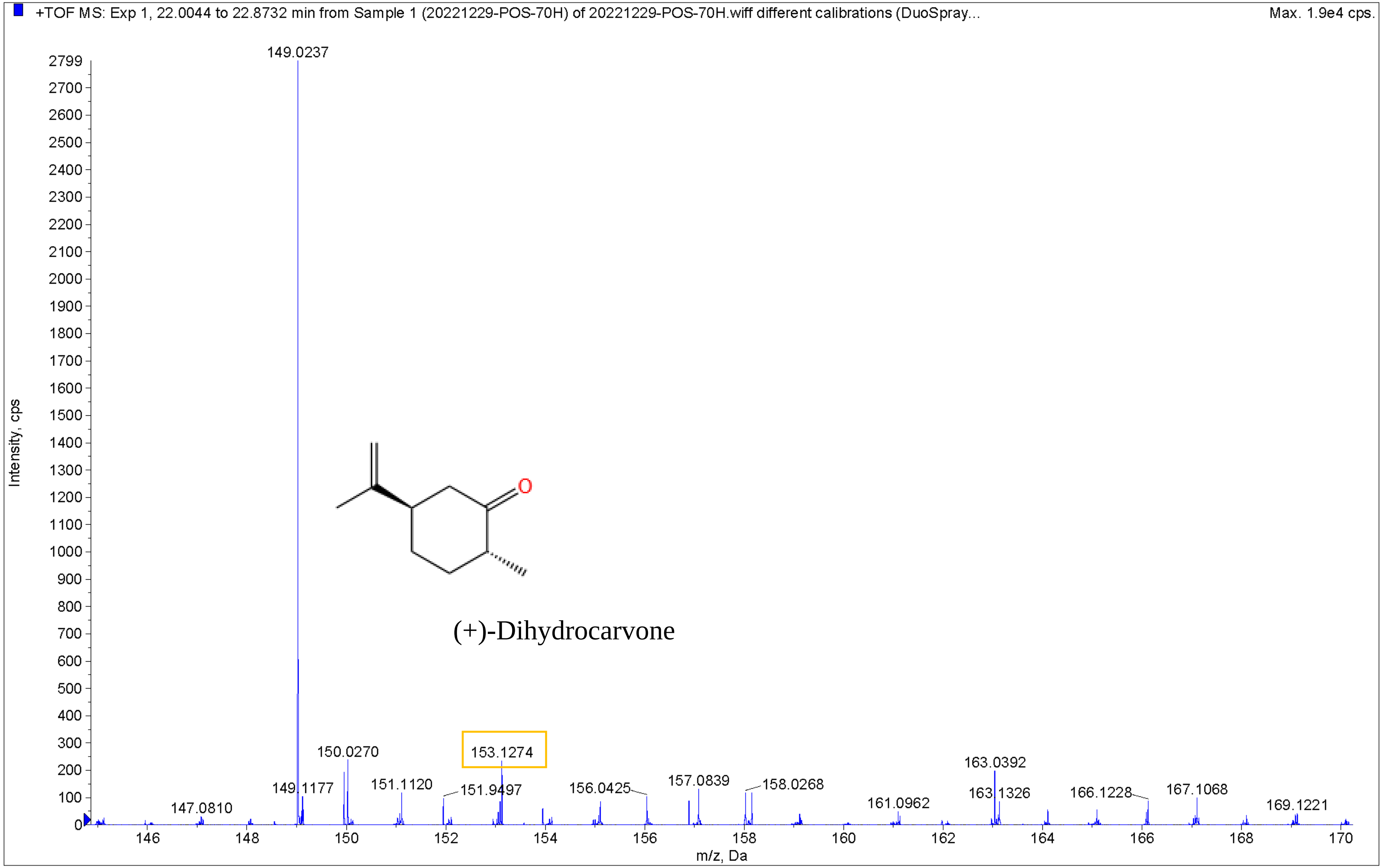

+TOF MS: Exp 1, 22.0044 to 22.8732 min from Sample 1 (20221229-POS-70H) of 20221229-POS-70H.wiff different calibrations (DuoSpray...
Max. 1.9e4 cps.
Intensity, cps
m/z, Da
149.0237
150.0270
153.1274
163.0392
149.1177
151.1120
151.9497
156.0425
157.0839
158.0268
161.0962
163.1326
166.1228
167.1068
169.1221
147.0810
O
(+)-Dihydrocarvone

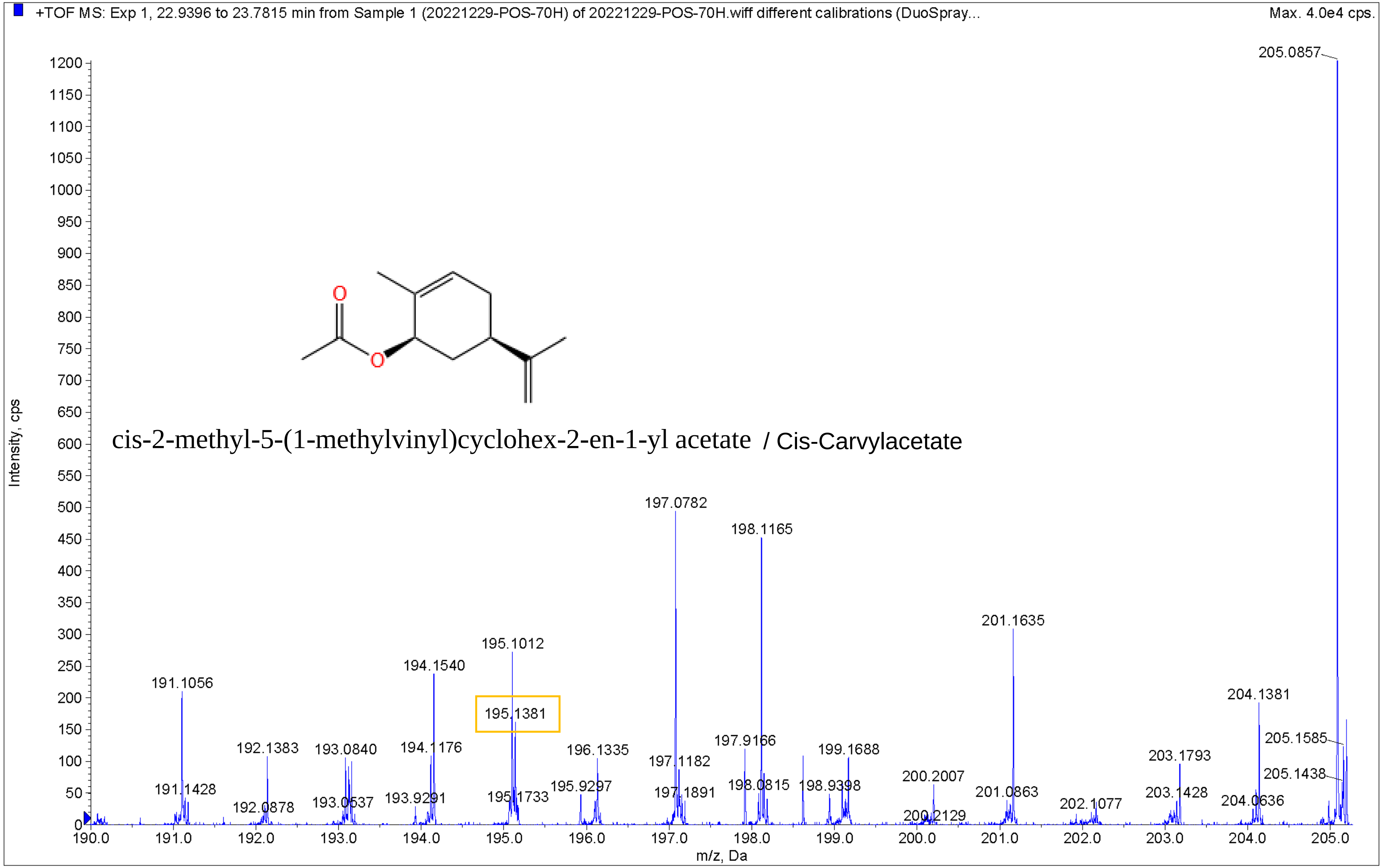
+TOF MS: Exp 1, 22.9396 to 23.7815 min from Sample 1 (20221229-POS-70H) of 20221229-POS-70H.wiff different calibrations (DuoSpray...
Max. 4.0e4 cps.
Intensity, cps
m/z, Da
cis-2-methyl-5-(1-methylvinyl)cyclohex-2-en-1-yl acetate / Cis-Carvylacetate
205.0857
197.0782
198.1165
201.1635
195.1012
194.1540
191.1056
204.1381
195.1381
205.1585
205.1438
203.1793
199.1688
197.9166
196.1335
194.1176
193.0840
192.1383
191.1428
197.1182
200.2007
201.0863
203.1428
204.0636
202.1077
200.2129
198.9398
198.0815
197.1891
195.9297
195.1733
193.9291
193.0537
192.0878

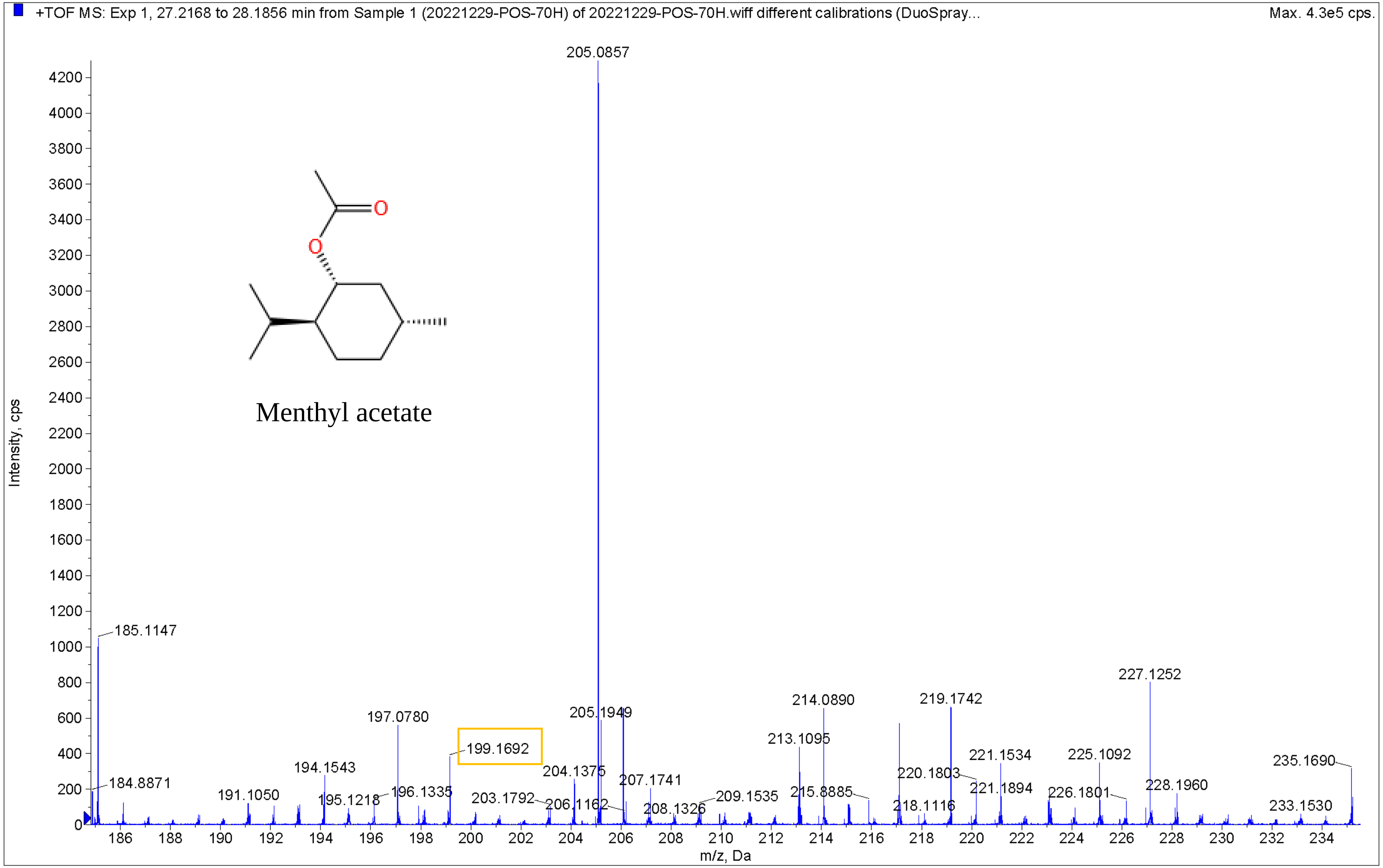

+TOF MS: Exp 1, 27.2168 to 28.1856 min from Sample 1 (20221229-POS-70H) of 20221229-POS-70H.wiff different calibrations (DuoSpray...
Max. 4.3e5 cps.
Intensity, cps
m/z, Da
Menthyl acetate
205.0857
185.1147
184.8871
191.1050
194.1543
195.1218
196.1335
197.0780
199.1692
203.1792
204.1375
205.1949
206.1162
207.1741
208.1326
209.1535
213.1095
214.0890
215.8885
218.1116
219.1742
220.1803
221.1534
221.1894
225.1092
226.1801
227.1252
228.1960
233.1530
235.1690

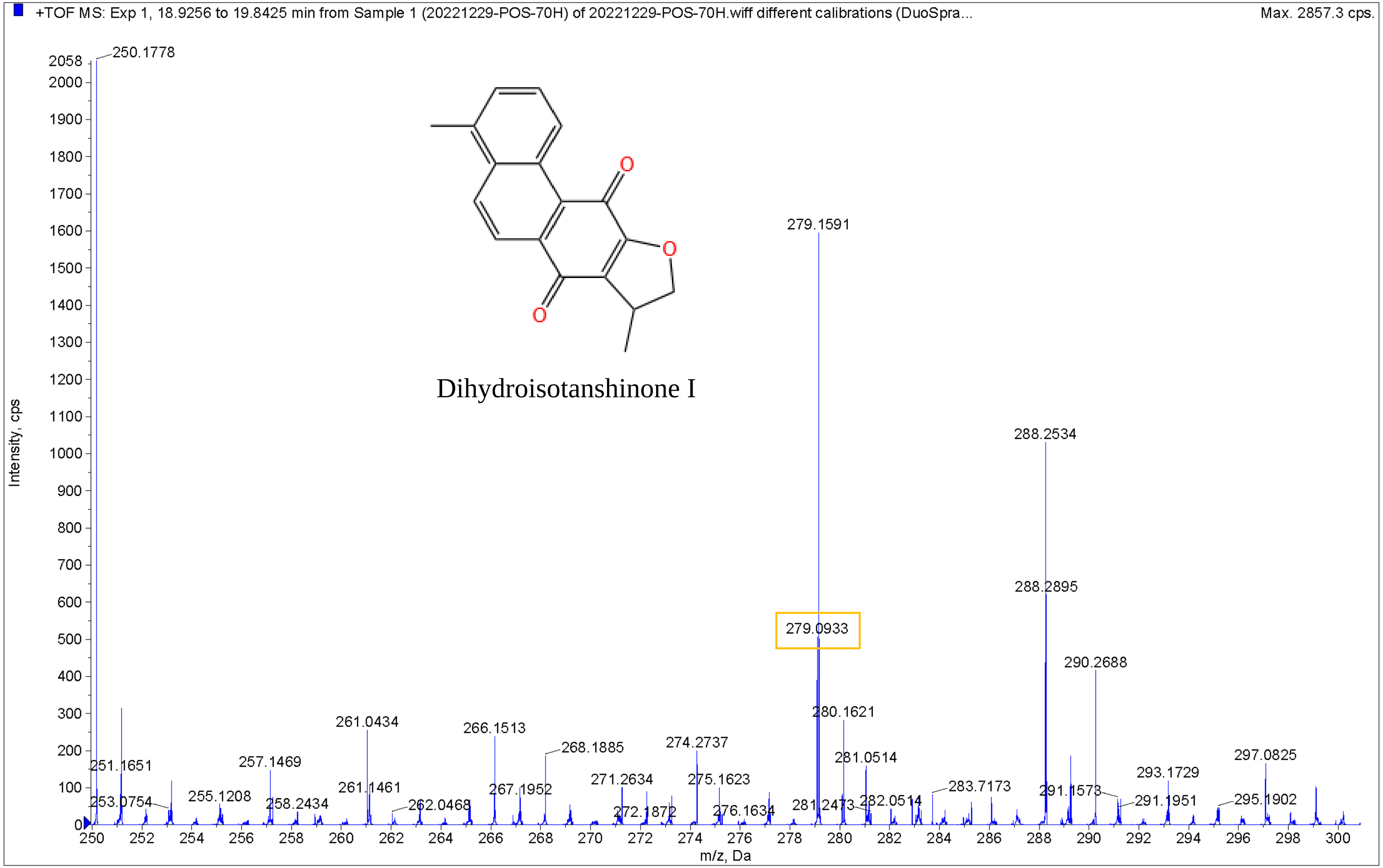

+TOF MS: Exp 1, 18.9256 to 19.8425 min from Sample 1 (20221229-POS-70H) of 20221229-POS-70H.wiff different calibrations (DuoSpra...
Max. 2857.3 cps.
Dihydroisotanshinone I
Intensity, cps
m/z, Da
250.1778
279.1591
288.2534
288.2895
279.0933
290.2688
280.1621
281.0514
261.0434
266.1513
268.1885
274.2737
297.0825
293.1729
251.1651
257.1469
271.2634
275.1623
283.7173
261.1461
267.1952
291.1573
291.1951
295.1902
253.0754
255.1208
258.2434
262.0468
272.1872
276.1634
281.2473
282.0514

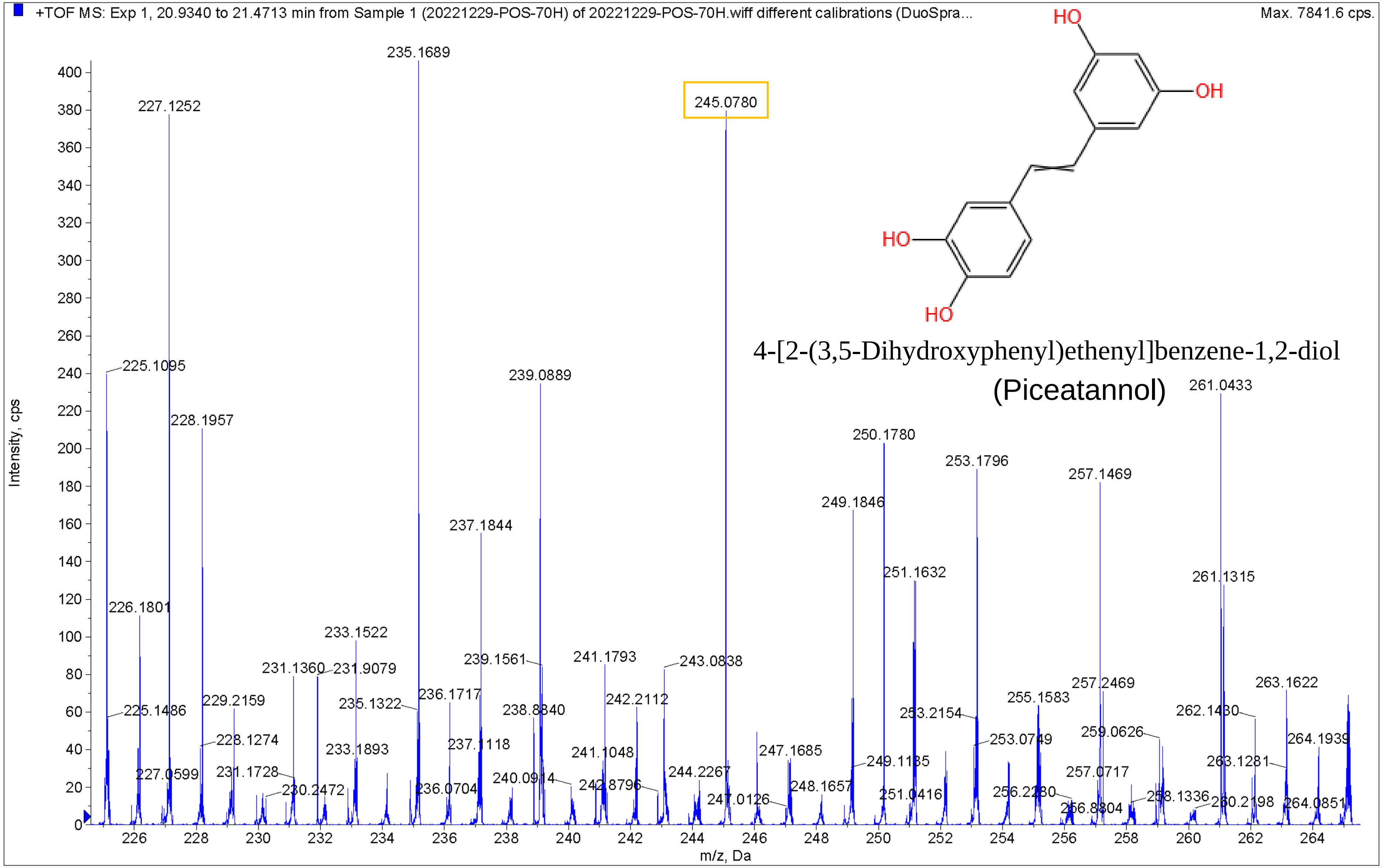
+TOF MS: Exp 1, 20.9340 to 21.4713 min from Sample 1 (20221229-POS-70H) of 20221229-POS-70H.wiff different calibrations (DuoSpra...
Max. 7841.6 cps.
Intensity, cps
m/z, Da
235.1689
227.1252
245.0780
225.1095
239.0889
228.1957
261.0433
250.1780
253.1796
257.1469
249.1846
237.1844
251.1632
261.1315
226.1801
233.1522
4-[2-(3,5-Dihydroxyphenyl)ethenyl]benzene-1,2-diol
(Piceatannol)
HO
OH
HO
HO

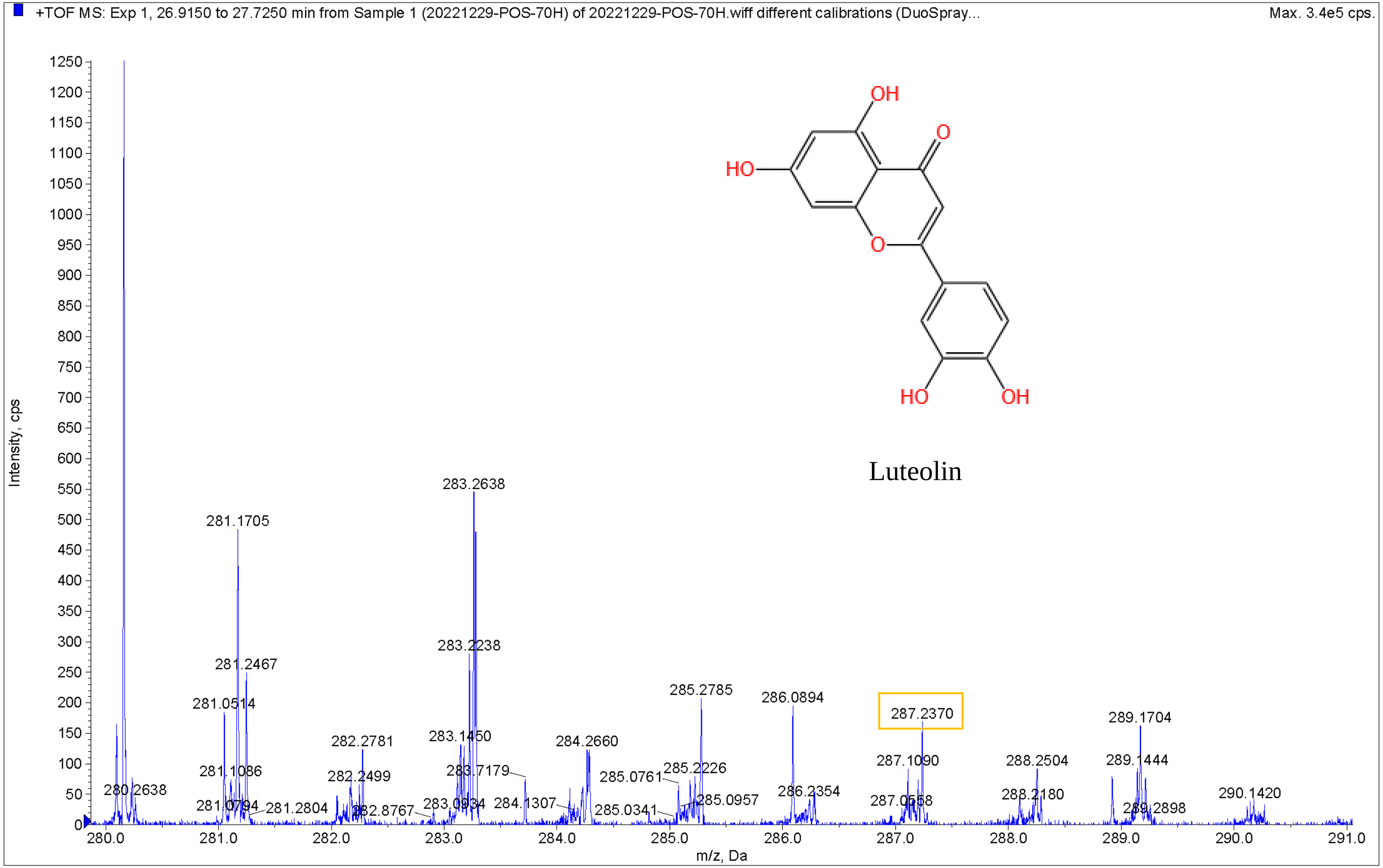
+TOF MS: Exp 1, 26.9150 to 27.7250 min from Sample 1 (20221229-POS-70H) of 20221229-POS-70H.wiff different calibrations (DuoSpray...
Max. 3.4e5 cps.
Intensity, cps
m/z, Da
281.1705
283.2638
283.2238
281.2467
281.0514
285.2785
286.0894
287.2370
289.1704
282.2781
283.1450
284.2660
287.1090
288.2504
289.1444
OH
O
HO
O
HO
OH
Luteolin

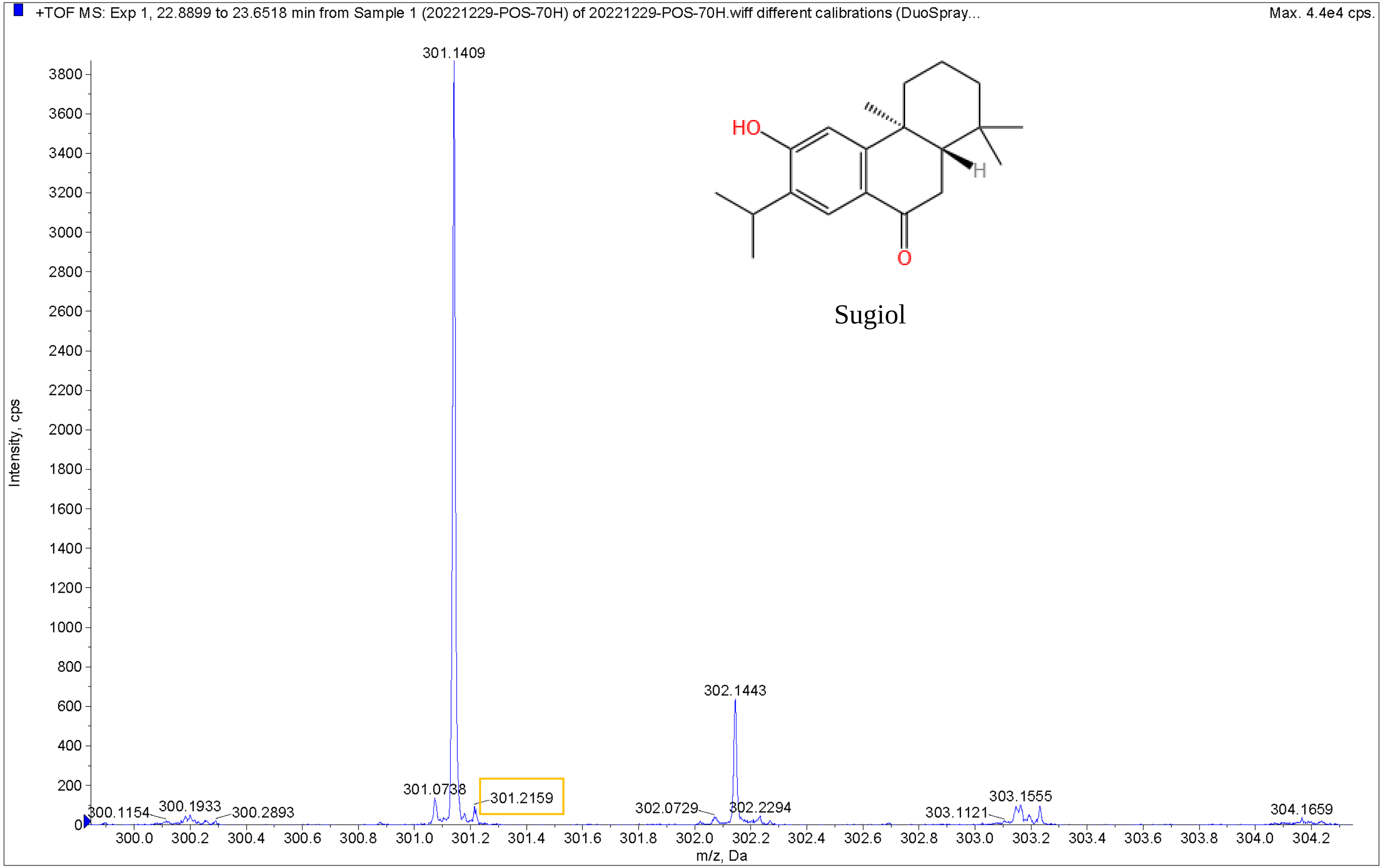
+TOF MS: Exp 1, 22.8899 to 23.6518 min from Sample 1 (20221229-POS-70H) of 20221229-POS-70H.wiff different calibrations (DuoSpray...
Max. 4.4e4 cps.
301.1409
302.1443
301.0738
301.2159
300.1154
300.1933
300.2893
302.0729
302.2294
303.1121
303.1555
304.1659
Intensity, cps
m/z, Da
HO
O
H
Sugiol

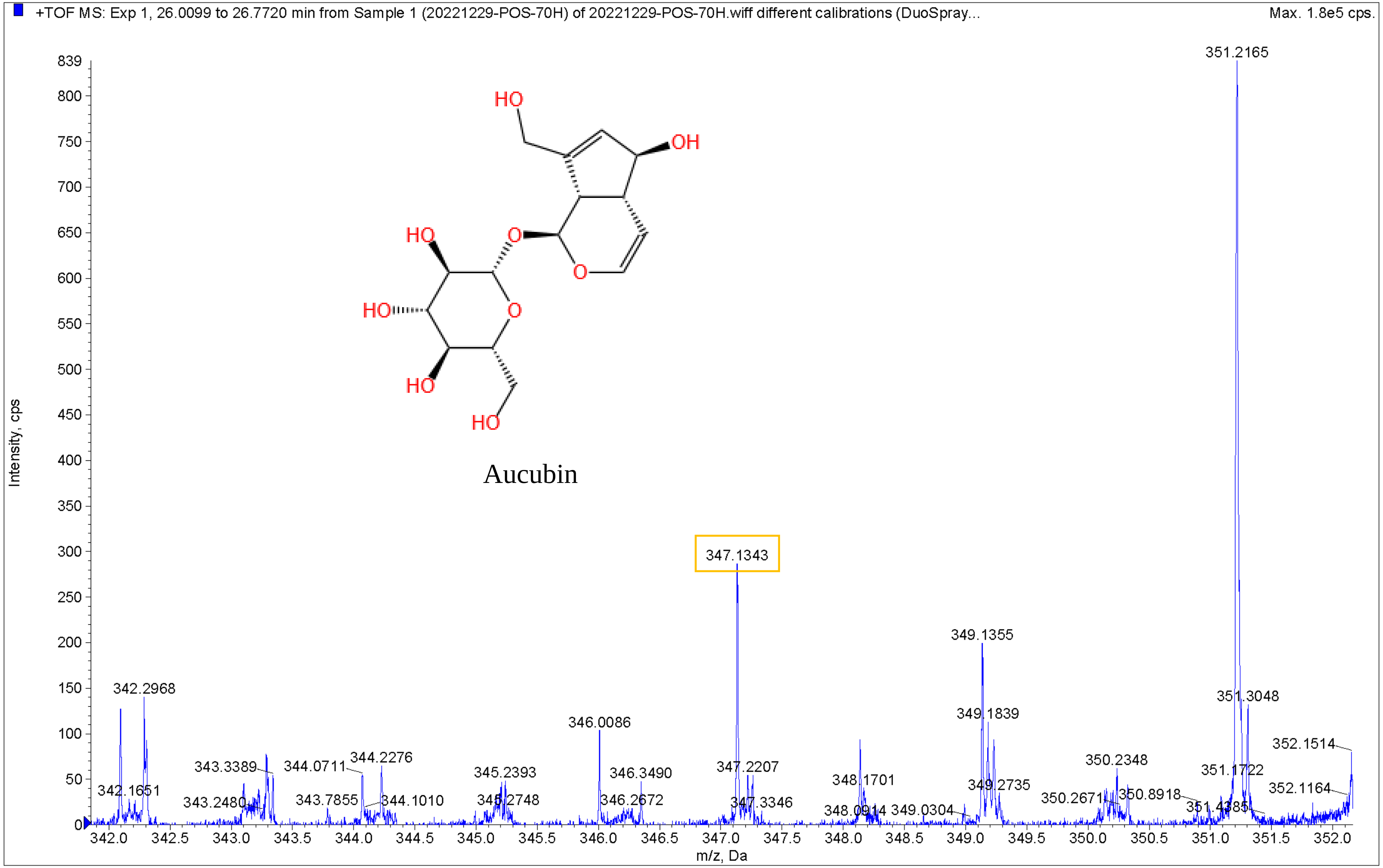
+TOF MS: Exp 1, 26.0099 to 26.7720 min from Sample 1 (20221229-POS-70H) of 20221229-POS-70H.wiff different calibrations (DuoSpray...
Max. 1.8e5 cps.
Intensity, cps
m/z, Da
Aucubin
HO
OH
O
HO
HO
HO
HO
351.2165
347.1343
349.1355
342.2968
346.0086
351.3048
349.1839
352.1514

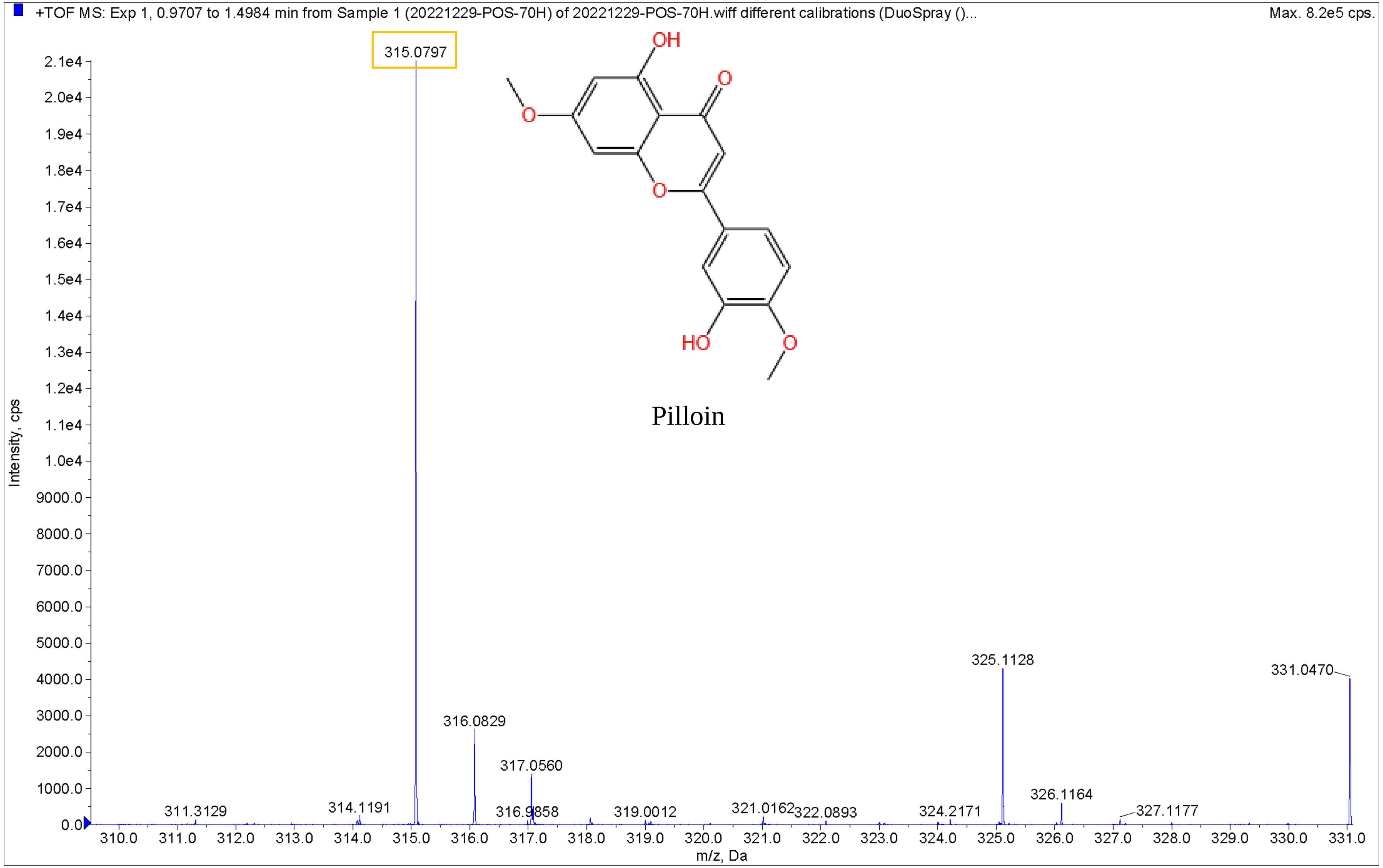

+TOF MS: Exp 1, 0.9707 to 1.4984 min from Sample 1 (20221229-POS-70H) of 20221229-POS-70H.wiff different calibrations (DuoSpray ()...
Max. 8.2e5 cps.
Intensity, cps
m/z, Da
315.0797
316.0829
317.0560
316.9858
311.3129
314.1191
319.0012
321.0162
322.0893
324.2171
325.1128
326.1164
327.1177
331.0470
OH
O
O
O
HO
O
Pilloin

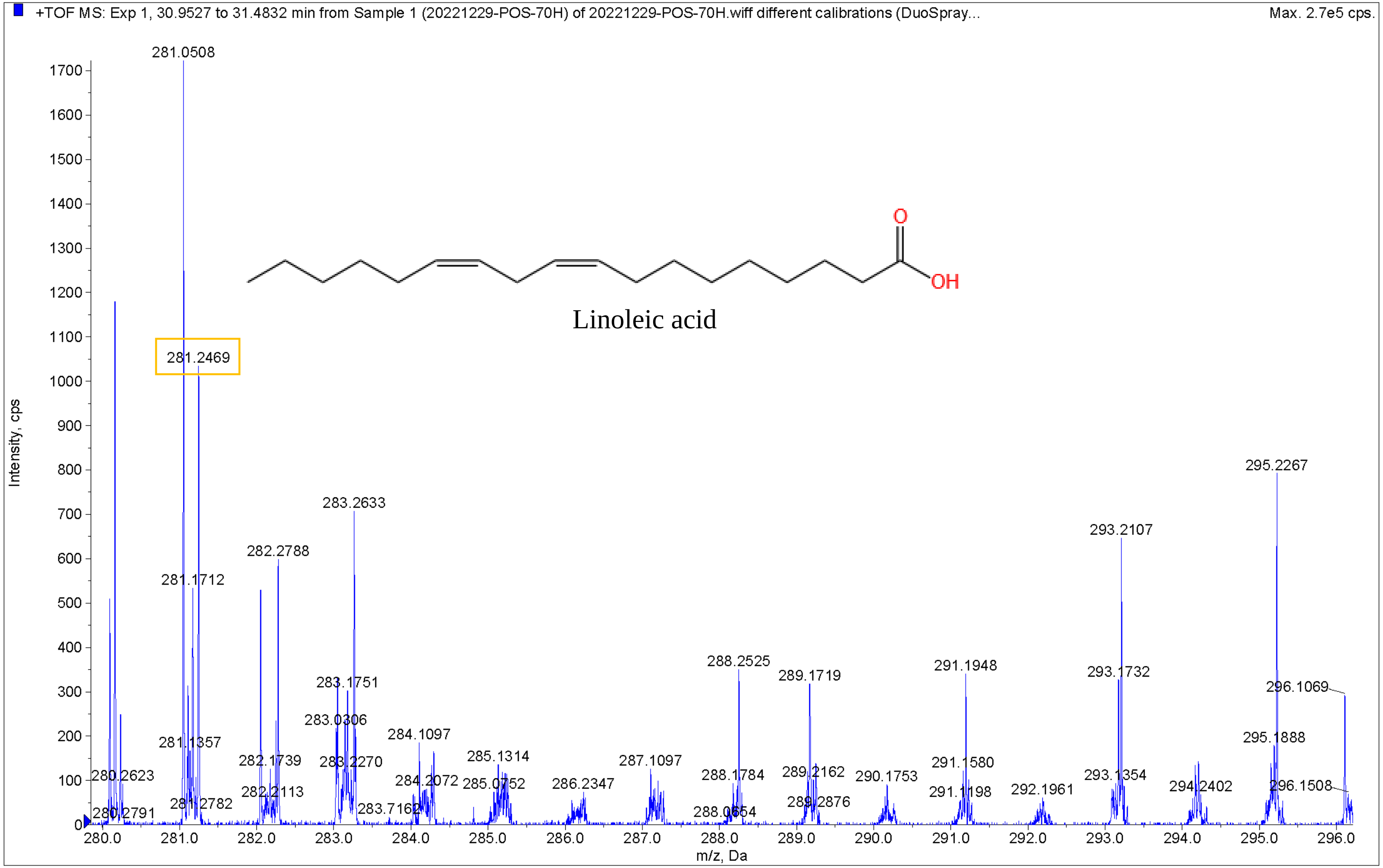

+TOF MS: Exp 1, 30.9527 to 31.4832 min from Sample 1 (20221229-POS-70H) of 20221229-POS-70H.wiff different calibrations (DuoSpray...
Max. 2.7e5 cps.
Intensity, cps
m/z, Da
Linoleic acid
O
OH
281.0508
281.2469
283.2633
282.2788
281.1712
295.2267
293.2107
288.2525
289.1719
291.1948
293.1732
296.1069

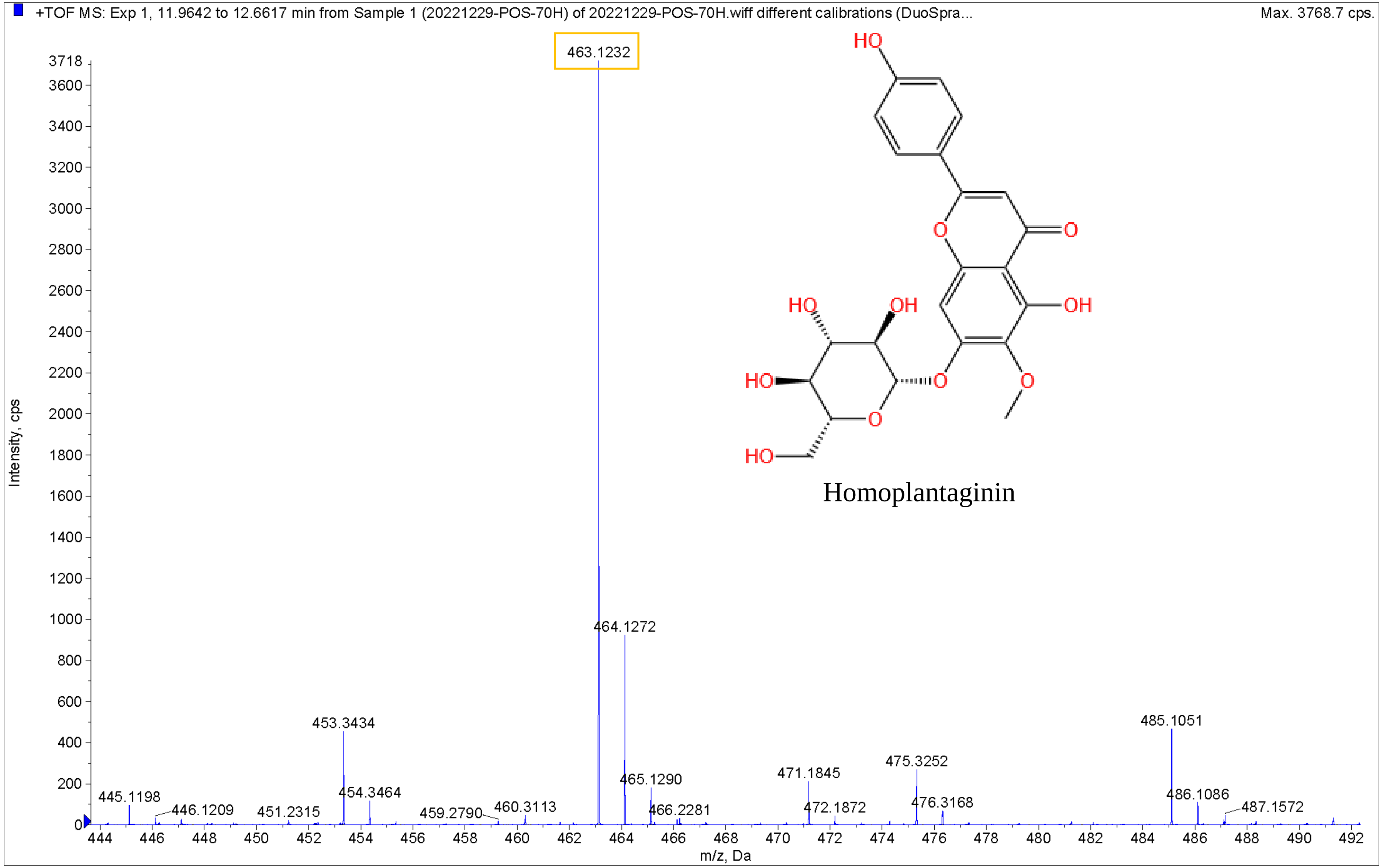
+TOF MS: Exp 1, 11.9642 to 12.6617 min from Sample 1 (20221229-POS-70H) of 20221229-POS-70H.wiff different calibrations (DuoSpra...
Max. 3768.7 cps.
Intensity, cps
m/z, Da
463.1232
464.1272
453.3434
485.1051
475.3252
471.1845
465.1290
454.3464
445.1198
446.1209
451.2315
459.2790
460.3113
466.2281
472.1872
476.3168
486.1086
487.1572
Homoplantaginin
